# Editorial: Optimizing the therapeutic potential in clinical settings: leveraging placebos and mitigating nocebo effects

**DOI:** 10.3389/fpsyg.2026.1834283

**Published:** 2026-05-07

**Authors:** Vanda Faria, Michael Bernstein, Marco Annoni, Tomas Furmark, Eric A. Moulton

**Affiliations:** 1 Department of Psychology, Uppsala University, Uppsala, Sweden; 2 Brain and Eye Pain Imaging Lab, Pain & Affective Neuroscience Center, Anesthesiology, Critical Care and Pain Medicine, Boston Children's Hospital and Harvard Medical School, Boston, MA, United States; 3 Department of Radiology, Warren Alpert Medical School, Brown University, Providence, RI, United States; 4 Rhode Island Hospital, Brown University Health, Providence, RI, United States; 5 National Research Council of Italy (CNR), Interdepartmental Center for Research Ethics and Integrity (CID-Ethics), Rome, Italy

**Keywords:** clinical setting, placebo, nocebo, optimizing therapies, clinical translation

Placebo and nocebo effects are not peripheral phenomena in healthcare: rather, they are embedded within therapeutic encounters. Despite decades of research elucidating their psychosocial and neurobiological mechanisms, however, their systematic translation into clinical practice remains limited. This Research Topic was designed to address this translational gap and answer several key questions: How can placebo effects be ethically and effectively leveraged in real-world care? How can nocebo effects be mitigated without compromising transparency and patient autonomy? In addition, how can clinicians and researchers find common ground in conceptualizing and measuring contextual influences? The 12 contributions included in this Research Topic advance this agenda by examining conceptual clarity, implementation strategies, patient perspectives, legal frameworks, communication practices, and neurobiological mechanisms.

## Clarifying context: bridging clinical practice and research

The Opinion article by Poulter et al. provided an important conceptual anchor for this Research Topic, addressing misunderstandings surrounding contextual effects in musculoskeletal pain. The authors highlighted four key sources of tension between clinicians and researchers: terminology, clinical relevance, beliefs and expectations, and statistical estimation methods. They argued that contextual factors are components of all therapeutic encounters and called for clearer definitions, improved reporting practices, and constructive interdisciplinary dialogue. Their reflections suggest that these phenomena should not be viewed as unusual problems to eliminate but as integral elements of clinical care that require thoughtful integration and rigorous investigation. This conceptual groundwork sets the stage for the empirical contributions that follow.

## Open-label placebos: translational pathways without deception

A central translational focus of this Research Topic is open-label placebos (OLPs), which aim to harness placebo effects while maintaining transparency. Paschke-Dahl and Klinger investigated OLPs in fibromyalgia patients undergoing gluten challenges using a 2 × 2 design (gluten vs. sham gluten and neutral vs. positive OLP instructions). Positive OLP instructions were associated with decreases in pain over time, while neutral OLP instructions were associated with increases. Gluten itself showed no significant effect on pain or indigestion, and a strong correlation was observed between expected and actual pain relief. These findings highlight the importance of expectation-related processes in symptom modulation in this context. Two secondary analyses further suggest that responsiveness to OLPs may depend on participants' psychological profiles and their individual characteristics. Frisaldi et al. reported that, among medical students, OLP intake (compared to no intervention) is associated with improved test performance among those with higher test anxiety and among students who adopted beneficial learning strategies. Similarly, Caliskan et al. found that, among patients with chronic low back pain, lower baseline pain catastrophizing predicts objective improvement in spinal motion velocity in the OLP plus treatment-as-usual group, whereas those with higher catastrophizing do not show this benefit. Together, these studies underscore the importance of examining psychological moderators when considering OLP implementation. Complementing these findings, Nascimento et al. explored patients' attitudes toward OLP implementation through qualitative focus groups involving individuals with chronic back pain, chronic migraines, or chemotherapy-induced nausea/emesis. Identified themes reflected interest in and ambivalence toward using OLPs. To the extent patients were willing to consider OLPs, trust and clinician credibility were prerequisites. These findings suggest that successful clinical translation depends not only on demonstrating efficacy but also on how OLPs are communicated and embedded within therapeutic relationships, consistent with prior work on the importance of perceived warmth and competence from a healthcare provider (Howe et al., 2019).

## Mitigating nocebo effects through optimized communication

Just as positive expectations can enhance outcomes, negative expectations can amplify adverse experiences. Addressing this challenge, Asan et al. presented a preregistered, double-blind, randomized, controlled clinical trial protocol designed to test whether optimized communication during informed consent for lumbar puncture can reduce headache-related impairment compared to standard communication. The protocol was motivated by evidence that risk disclosure can increase reports of side effects by enhancing negative expectations. Importantly, the study aimed to mitigate potential nocebo-associated effects without withholding critical information from patients, thereby aligning ethical transparency with symptom prevention. This contribution exemplifies the practical and ethical complexities at the heart of placebo and nocebo translation: improving outcomes while preserving patient autonomy and trust.

## Legal and regulatory landscapes of clinical placebo use

The clinical application of placebos also unfolds within legal and regulatory frameworks. In “A Dose of Doubt,” Richard, Ganz, Elger et al. reported qualitative findings from Swiss primary care physicians, who described using impure placebos in practice and emphasized risk–benefit evaluation as a primary justification. Participants' knowledge of regulations varied, and they generally did not perceive a strong need for additional formal regulation. In a complementary legal analysis, “Noble Humbug?”, Richard, Ganz, Hornstein et al. examined binding and non-binding laws in France, Germany, Switzerland, the United Kingdom, and the United States. They reported that placebo use is often indirectly regulated through informed consent statutes rather than placebo-specific legislation and that professional guidelines, such as those from the American Medical Association, outline the conditions under which placebo use may be permissible. The authors emphasize that unauthorized placebo use may carry legal implications, highlighting the importance of clear standards and transparent communication. The emergence of OLPs is discussed as a development with the potential to address ethical tensions while preserving clinical flexibility.

## Enriched therapeutic encounters in interdisciplinary pain care

The importance of therapeutic context is further reflected in the retrospective study by Vora et al., which examined the outcomes of an interdisciplinary functional restoration program for adults with high-impact chronic pain and clinically elevated catastrophizing. Participants demonstrated statistically significant improvements in functional performance, satisfaction with function, pain ratings, mood, and catastrophizing following program completion. The authors describe the program's emphasis on collaboration, validation, trust, self-efficacy, and positive expectations. These findings align with broader themes in this Research Topic regarding the clinical relevance of enriched therapeutic encounters.

## Neurobiological and expectancy-based mechanisms

The Perspective by Furmark et al. revisited debates surrounding SSRIs and placebo effects in social anxiety disorder. The authors reported findings from neuroimaging studies in which SSRI and placebo responders show equal reductions in amygdala activity alongside comparable symptom improvements. They also discussed evidence from expectation manipulation and open–hidden administration designs, suggesting that overt SSRI administration yields greater symptom reduction than covert administration. Furthermore, serotonin transporter occupancy did not correlate with symptom improvement, underscoring the complexity of pharmacological and expectancy-related mechanisms. These findings challenge the idea that SSRIs are effective solely due to their serotonergic activity and underscore the importance of the interplay between neurobiology and psychological expectation in treatment response. Extending expectancy research into affect regulation, Schäfer and Rief experimentally investigated differently framed deceptive placebo instructions (antecedent-focused vs. response-modulating) in healthy volunteers following shame induction. Both placebo framings influenced changes in state shame, rumination, and cognitive flexibility relative to control. Personality traits, including experiential avoidance and emotional control, modulated responses in the antecedent-focused condition. This work expands the scope of placebo research beyond symptom relief to cognitive and affective processes.

## Emerging contexts: technology and the therapeutic relationship

Finally, Faria et al. explored physicians' perspectives on the integration of generative artificial intelligence into healthcare. A majority of surveyed physicians had used genAI tools, primarily for administrative tasks, and many believed that these tools could improve patient care or increase time for direct interaction. However, responses were more divided regarding empathy and placebo-related processes, and over half of the physicians agreed that genAI-human interactions could increase patient anxiety. As digital tools increasingly shape clinical workflows, these findings highlight the importance of examining how technological mediation may interact with the relational and expectation-related dimensions of care.

## Conclusion

Across diverse methodologies and clinical contexts, the contributions in this Research topic converge on a shared message: placebo and nocebo effects are not topics of mere academic curiosity but are clinically relevant processes embedded within communication, expectations, interpersonal dynamics, and healthcare systems. They play both direct and indirect roles in patient outcomes. The studies presented here advance our understanding of when and for whom OLP approaches may be beneficial, how communication strategies might reduce nocebo-associated harms, how legal frameworks shape practice, the potential benefits and consequences of AI integration with patient care in these domains, and how neurobiological and psychological mechanisms interact. Bridging experimental insight and clinical implementation remains a complex task. However, by integrating conceptual clarifications, empirical analyses, qualitative perspectives, communication protocols, and legal scholarship, this Research Topic takes meaningful steps toward optimizing the therapeutic potential of placebo and nocebo effects in clinical settings, leveraging beneficial expectancy effects while mitigating unintended harms.
